# Combination therapy of BCR-ABL-positive B cell acute lymphoblastic leukemia by tyrosine kinase inhibitor dasatinib and c-JUN N-terminal kinase inhibition

**DOI:** 10.1186/s13045-020-00912-3

**Published:** 2020-06-18

**Authors:** Xinhua Xiao, Ping Liu, Donghe Li, Zhizhou Xia, Peihong Wang, Xiuli Zhang, Mingzhu Liu, Lujian Liao, Bo Jiao, Ruibao Ren

**Affiliations:** 1 Shanghai Institute of Hematology, State Key Laboratory of Medical Genomics, National Research Center for Translational Medicine at Shanghai, Collaborative Innovation Center of Hematology, Ruijin Hospital Affiliated to Shanghai Jiao Tong University School of Medicine, Shanghai, China, Ruijin Hospital, Shanghai Jiao Tong University School of Medicine, Shanghai, China; 2 Shanghai Key Laboratory of Regulatory Biology, School of Life Sciences, East China Normal University, Shanghai, China; 3 Department of Biology, Brandeis University, Waltham, MA USA

**Keywords:** Ph^+^ B-ALL, JNK, Dasatinib, Combination target therapy

## Abstract

**Background:**

The Philadelphia chromosome (Ph), which leads to the creation and expression of the fusion gene product BCR-ABL, underlines the pathogenesis of chronic myelogenous leukemia (CML) and a fraction of adult and pediatric acute B-lymphoblastic leukemia (B-ALL). The BCR-ABL tyrosine kinase inhibitors (TKIs) have shown a remarkable clinical activity in patients with CML, but their efficacy in treating Ph^+^ B-ALL is limited. Identifying additional therapeutic targets is important for the effective treatment of Ph^+^ B-ALL.

**Methods:**

Activation of the JNK signaling pathway in human and mouse BCR-ABL^+^ B-ALL cells with or without dasatinib treatment was analyzed by Western blotting. JNK was inhibited either by RNA interference or chemical inhibitors, such as JNK-IN-8. The effect of JNK inhibition with or without BCR-ABL TKI dasatinib on BCR-ABL^+^ B-ALL cells was analyzed by the CellTiter-Glo® Luminescent Cell Viability Assay. The in vivo effects of JNK-IN-8 and dasatinib alone or in combination were tested using a BCR-ABL induced B-ALL mouse model.

**Results:**

We found that the c-JUN N-terminal kinase (JNK) signaling pathway is abnormally activated in both human and mouse BCR-ABL^+^ B-ALL cells, but the BCR-ABL TKI does not inhibit JNK activation in these cells. Inhibition of JNK, either by RNAi-mediated downregulation or by JNK inhibitors, could significantly reduce viability of Ph^+^ B-ALL cells. JNK inhibition by RNAi-mediated downregulation or JNK inhibitors also showed a synergistic effect with the BCR-ABL TKI, dasatinib, in killing Ph^+^ B-ALL cells in vitro. Furthermore, a potent JNK inhibitor, JNK-IN-8, in combination with dasatinib markedly improved the survival of mice with BCR-ABL induced B-ALL, as compared to the treatment with dasatinib alone.

**Conclusions:**

Our findings indicate that simultaneously targeting both BCR-ABL and JNK kinase might serve as a promising therapeutic strategy for Ph^+^ B-ALL.

## Background

The t(9;22)(q34;q11) reciprocal translocation that creates a minute chromosome, known as the Philadelphia chromosome (Ph), is a hallmark of chronic myelogenous leukemia (CML) and also present in about 3% of pediatric B-ALL and 25% of adult B cell acute lymphoblastic leukemia (Ph^+^ B-ALL) [1]. The translocation leads to the creation and expression of the fusion gene product BCR-ABL. The ABL tyrosine kinase is activated in the BCR-ABL fusion protein and plays a central role in the pathogenesis of CML and Ph^+^ B-ALL [2].

Imatinib mesylate, a selective ABL tyrosine kinase inhibitor (TKI), has shown a remarkable clinical activity in patients with CML [3]. Significant therapeutic effects and clinical benefits have been achieved in treating Ph^+^ B-ALL, especially when combined with hematopoietic stem cell transplantation and chemotherapy [4]. However, relapse of Ph^+^ B-ALL remains a clinical problem. The second-generation ABL TKI dasatinib could yield remarkable responses in the treatment of imatinib-resistant Ph^+^ B-ALL patients by co-targeting BCR-ABL and SRC family kinases [5, 6]. Unfortunately, eventual relapse seems inevitable when leukemia cells reemerge in bone marrow with additional genetic variations and rewired survival and proliferative signaling [7–9]. Besides, severe side effects such as cardiotoxicity [10] and pulmonary hypertension [11] are more likely to be induced by dasatinib. Thus, novel therapeutic targets are needed to treat Ph^+^ B-ALL more effectively.

Mitogen-activated protein kinase (MAPK) signaling pathways play a crucial role in the regulation of tumor cell growth, proliferation, migration, and apoptosis [12]. MAPKs belong to a diverse family of serine/threonine protein kinases, including four major subfamilies, such as c-JUN N-terminal kinase (JNK), p38 MAP kinase, extracellular signal-regulated kinase (ERK) 1/2, and ERK5 [13]. Among them, JNK signaling is a unique MAPK pathway that is predominantly activated in cells under the stress conditions such as ROS production and inflammation [14]. There are three JNK homologs in mammalians, JNK1/2/3, which are encoded by *MAPK8*/*9*/*10* genes, respectively [15]. JNK1/2 are constitutively expressed in almost all tissues, while JNK3 restricts in brain, heart, and testis [16]. JNK activation is through phosphorylation by MAPK kinases MKK4 and MKK7 [17] and the activation of JNK plays an important role in cell survival, cell proliferation, cell differentiation [14, 17], and cancer stem cell maintenance [18]. BCR-ABL protein significantly activates the JNK signaling pathway in transformed cells [19, 20]. More importantly, depletion of *Mapk8* mitigates the BCR-ABL-induced transformation in mouse B lymphoblasts and prolongs the survival of mice with BCR-ABL induced B-ALL [21]. However, it is not clear how important is the JNK activation in the maintenance of Ph^+^ B-ALL and whether the JNK inhibition could cooperate with BCR-ABL inhibitors in treating Ph^+^ B-ALL.

In this study, using both BCR-ABL induced B-ALL mouse model and human B-ALL cells, we found that the activation of JNK could not be inhibited by BCR-ABL TKI in B-ALL cells. Targeting JNK by either RNA interference or chemical inhibitors decreased the cell viability of Ph^+^ B-ALL. The JNK inhibitor and BCR-ABL TKI dasatinib could synergistically kill Ph^+^ B-ALL cells in vitro and greatly improve the survival of mice with BCR-ABL induced B-ALL.

## Material and method

### Cell lines and cell culture

SUP-B15 and K562 cell lines were purchased from ATCC and cultured in RPMI 1640 (Basal Media, China) supplemented with 10% fetal bovine serum (FBS, Moregate, Batch No. 827106). Cell line identities were validated by using short tandem repeat profiling analysis according to the American National Standard ANS-0002-2011 at the laboratory of VivaCell Bioscience Co. The cell passages were limited to 15 generations for all experiments in this study. Mycoplasma contamination was excluded using the antibiotics Mycoplasmincin (InvivoGen) and periodically examined using MycoFluor Mycoplasma Detection Kit (Invitrogen, #M7006).

### Magnetic-activated cell sorting

BM cells extracted from BALB/cByJ mice were incubated with CD19 antibody conjugated microbeads (Miltenyi Biotec, #130-097-144) for 30 min and enriched by MACS separators per manufacture’s instruction.

### Flow cytometry-based cell sorting and analysis

Cells from mouse peripheral blood and BM were firstly lysed with red blood cell lysis buffer and then labeled by antibodies against Mac-1-PE (Bio legend, #101208) and CD19-APC (BD Biosciences, #550992) in staining buffer (PBS, 1% FBS). After staining in dark for 15 min at room temperature, samples were washed with PBS and resuspended in staining buffer. Flow cytometry analysis and sorting were performed on an LSR II system (BD Biosciences). The cell population with given surface markers were analyzed by FlowJo software. Human cell line SUP-B15 stably infected with shJNK#1, #2, or NC were sorted based on GFP expression.

### Generation of lentiviruses and retroviruses

Two distinct shRNA oligonucleotides were designed for knocking down JNKs, of which sequences are described as following: ShJNK#1 sense (TGAAAGAATGTCCTACCTTCT) and antisense (AGAAGGTAGGACATTCTTTCA); ShJNK#2 sense (GCAGAAGCAAGCGTGACAACA) and antisense (TGTTGTCACGCTTGCTTCTGC). Paired oligonucleotides were annealed and inserted into lentiviral expression vectors (pLKO.1-GFP). The JNK-targeted or scrambled non-specific control (NC) shRNA plasmids were co-transfected with the lentiviral packaging vectors, psPAX2 and pMD2G, using Lip6000 reagent (Beyotime Biotechnology) in 293 T cells to produce shJNK#1, #2, or NC lentiviruses. Likewise, BCR-ABL^p190^ retroviruses were produced using MSCV-BCR/ABL^p190^-IRES-GFP retroviral constructs as described previously [22].

### Bone marrow transduction and transplantation mouse model

The mouse bone marrow transduction/transplantation model for Ph^+^ B-ALL was established as described previously [22]. Briefly, bone marrow (BM) cells or CD19^+^ B lymphocytes isolated from the bone marrow of 6- to 8-week-old BALB/cByJ donor mice using MACS were transduced with BCR-ABL retroviruses suspended in a cocktail medium containing 50% (vol/vol) BCR-ABL retroviral supernatant, DMEM, 5% FBS, 100 U/mL penicillin, 100 μg/mL streptomycin, 5% WEHI-3-conditioned medium, 10 ng/mL IL-7, and 8 μg/mL Polybrene (Sigma Aldrich) by spinoculation at 1200×*g* for 90 min, followed by incubation at 37 °C for additional 4.5 h. Following the retroviral transduction, BM cells were washed with serum-free media and transplanted into sublethally irradiated (450 cGy) syngenic recipient mice, 10^6^ cells per mouse, via tail vein. All animal experiments were approved by The Animal Care & Welfare Committee of Ruijin Hospital affiliated to Shanghai Jiao Tong University School of Medicine.

### Cell viability assay

Cell viability assays were carried out using the CellTiter-Glo® Luminescent Cell Viability Assay as previously described [23]. Cells were seeded into 96-well cell culture plates at a density of 5000 cells per well and added with indicated drugs at various concentrations. After 48 h incubation, cells were lysed by CellTiter Glo reagent (Promega, #G7573), and the luminescence signals produced by ATP molecules from live cells were measured using an Envision plate reader (PerkinElmer) after 30 min incubation at room temperature.

### Western blot analysis

Western blot analysis was performed as previously described [24]. In brief, cell samples were counted and lysed in 1× sodium dodecyl sulfate (SDS) sample loading buffer. Equal amount of protein samples was loaded on polyacrylamide gel, followed by transfer to nitrocellulose membrane. The membrane was then blotted with specific primary antibodies against p-c-Abl (Y412, #2865S), BCR (#3902S), p-stat5 (Y694, #9351S), stat5 (#9363S), AKT (#4685S), p-AKT (Ser473, #4060S), ERK (#49655S), p-ERK (T202/Y204, #4370S), p-JNK (T183/Y185, #4668S), p-c-JUN (Ser63, #2361S) (all purchased from Cell Signaling Technology), c-Myc (#ab32072, Abcam), BRD4 (#ab128874, Abcam), JNK (#66210-1-Ig), c-JUN(#66313-1-Ig), and actin-HRP (#HRP-60008) (purchased from Proteintech). After overnight incubation at 4 °C, HRP-conjugated secondary antibodies were applied and luminescence signals on membrane were detected with electrochemical luminescence (Shanghai Share-bio Biotechnology). The western blot images were taken with a LAS4000 imaging system (Fujifilm).

### Patient sample preparation

Heparinized bone marrow samples were collected from 6 patients with newly diagnosed Ph^+^ B-ALL (detailed information for these patients are provided in Supplementary Table S2). Mononuclear cells (MNCs) were then separated by density gradient centrifugation using Lymphoprep reagent (Stemcell Technologies). Subsequently, MNCs were cultured in StemSpan basic media (Stemcell Technologies) supplemented with 10 ng/mL human stem cell factor, 10 ng/mL human IL-3, 10 ng/mL human IL-6 (all above cytokines were purchased from R&D Systems), 100 U/mL penicillin, and 100 μg/mL streptomycin (both from BBI Life Sciences). This study was approved by the Institutional Review Board of the Ruijin Hospital affiliated to Shanghai Jiao Tong University School of Medicine. Informed consent for the in vitro drug testing studies was obtained in accordance with the Declaration of Ruijin Hospital affiliated to Shanghai Jiao Tong University School of Medicine.

### Compounds and in vivo drug testing

JNK-IN-8, SP600125, JQ-1, imatinib, and dasatinib were all purchased from Selleck Chemicals. For the treatment of BCR-ABL^+^ B-ALL mouse model, mice were randomly separated into four groups, for comparing the effects of the vehicle control, dasatinib, JNK-IN-8, and both drugs in combination. Drugs or vehicle was administered after 10 days post BMT. The consecutive treatment was conducted in the following 18 days. Dasatinib was dissolved in 80 mM sodium citrate (pH 3.1) and administrated through oral gavage at the dosage of 2 mg/kg/day. JNK-IN-8 was dissolved successively in 2% DMSO, 30% PEG 300, 5% Tween 80, and deionized water and injected intraperitoneally at 20 mg/kg once a day. To monitor leukemia progress in mice, peripheral blood was collected via retro-orbital plexus and analyzed by using a pocH-100iV Diff hemocytometer (Sysmex Corporation) and flow cytometer. After 10 days post treatment, representative mice were sacrificed and autopsied, and samples from selected organs were obtained and stained with hematoxylin and eosin (H&E).

### KiNativ profiling

SUP-B15 cells were treated with DMSO or 0.3 μM of imatinib or dasatinib for 2 h and cell lysates were extracted to detect kinase activity using Pierce™ Kinase Enrichment Kits and ActivX™ Probes according to the manufacturer’s instruction (Thermo scientific). Samples were analyzed by Q-Exactive liquid chromatography-mass spectrometry-tandem mass spectrometry with a linear ion trap mass spectrometer (LC-MS/MS, Thermo Scientific).

### Statistical analysis

GraphPad Prism 7 software was used for statistical data analysis. Two-tailed unpaired Student’s *t* test was used for mean comparison between two groups, whereas the Kaplan-Meier survival curve and log-rank test were used for survival analysis. Calcusyn v2.0 software was used for calculating the “combination index” (CI) of the drug combination treatment to depict synergism (CI < 1), addictive effect (CI = 1), or antagonism (CI > 1) [25]. *P* values < 0.05 were considered statistically significant, and different levels were denoted as *P < 0.05, **P < 0.01, and ***P < 0.001, respectively.

## Results

### Dasatinib is incapable of inhibiting JNK signaling abnormally activated in Ph^+^ B-ALL cells

**Fig. 1:**
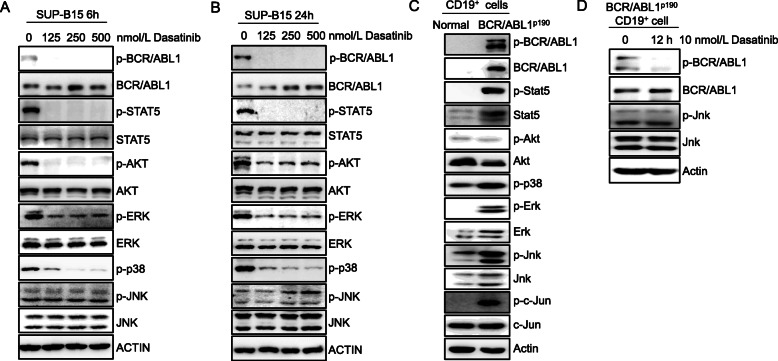
JNK kinases remain abnormally activated upon the treatment of dasatinib in Ph^+^ B-ALL. **a** Human Ph^+^ B-ALL cell line SUP-B15 cells were treated with dasatinib at various concentrations for 6 hours (h) and subsequently examined by western blot using indicated antibodies against key effectors in BCR-ABL and MAPK signaling pathways: phosphorylated (p) and total BCR/ABL, Stat5, AKT, ERK, p38 and JNK, respectively. Actin was used as a loading control. **b** SUP-B15 cells were treated with dasatinib at various concentrations as indicated for 24 h and subsequently examined by western blot using indicated antibodies against phosphorylated and total BCR/ABL, Stat5, AKT, ERK, p38, and JNK, respectively. Actin was used as a loading control. **c** Western blot analysis of lysates of CD19^+^ B lymphocytes isolated from bone marrows of normal and BCR/ABL^p190^ bone marrow transduction and transplantation mice using antibodies described in (**a**) plus western blot analysis using antibodies against phosphorylated and total c-JUN. **d** CD19^+^ B lymphocytes from BCR/ABL^p190^ bone marrow transduction and transplantation mice were treated with 10 nmol/L dasatinib or vehicle for 12 h, followed by western blot analysis with antibodies against phosphorylated and total BCR-ABL and JNK

To investigate the role of potential signaling pathways in sustaining Ph^+^ B-ALL cells upon the treatment of ABL TKI, we treated a widely used human Ph^+^ B-ALL cell line SUP-B15 with dasatinib for 6 h and confirmed that the phosphorylation of the BCR-ABL, as well as its classic downstream STAT5 [26] and AKT, was inhibited (Fig. 1a). In addition, the phosphorylation of MAPK kinase proteins ERK and p38 was also markedly suppressed (Fig. 1a). However, the phosphorylation of JNK remains unchanged (Fig. 1a). The same results were observed in SUP-B15 cells treated with dasatinib for 24 h (Fig. 1b). Moreover, similar to SUP-B15 cells, dasatinib is not able to inhibit phosphorylation of JNK in K562 cells, a Ph^+^ CML blast phase cell line, nor in patient-derived Ph^+^ B-ALL cells (Supplementary Fig. S1a, b, c, d, e and f). The expression of p-JNK was actually increased in SUP-B15 cells and four patient-derived Ph^+^ B-ALL cells after the dasatinib treatment (Supplementary Fig. S1a, b, c, d, e and f), indicating that JNK may be the key signaling for dasatinib resistance in Ph^+^ B-ALL.

We further checked the effect of dasatinib on JNK activation in a BCR-ABL^+^ B-ALL mouse model generated by transplantation of BCR-ABL^P190^-transduced CD19^+^ bone marrow cells (BMCs) into lethally irradiated recipient mice (Supplementary Fig. S2a and b). We found that JNK and phosphorylation of Stat5, ERK, JNK, and p38 are significantly upregulated in mouse BCR-ABL^+^ B-ALL cells (Fig. 1c). Similar to the results in human Ph^+^ B-ALL cell lines, the phosphorylation of JNK was not inhibited by dasatinib in mouse BCR-ABL^+^ B-ALL cells (Fig. 1d). Furthermore, we demonstrated that the JNK kinase activity is not affected by dasatinib or imatinib through KiNativ profiling [27] (Supplementary Fig. S2c and Supplementary table 1). These findings indicate that dasatinib does not affect the JNK signaling pathway that is abnormally activated in Ph^+^ B-ALL cells.

### JNK plays a critical role in the maintenance of Ph^+^ B-ALL cells but not CML cells

**Fig. 2:**
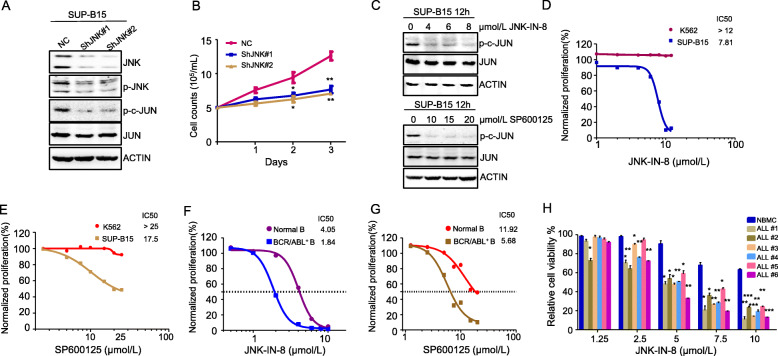
JNK inhibition reduces viability of Ph^+^ B-ALL cells. **a** Levels of phosphorylated and total JNK and c-JUN in SUP-B15 cells transduced with lentivirus vectors containing control shRNA (ShNC), shRNA targeting JNK (ShJNK#1), or ShJNK#2 were detected by western blot analysis with antibodies indicated. Actin was used as a loading control. **b** Proliferation of SUP-B15 cells expressing control and JNK shRNA were plotted by counting viable cells over a period of 3 days. **c** SUP-B15 cells were treated with JNK inhibitor JNK-IN-8 or SP600125 for 12 h, followed by western blot analysis using antibodies against phosphorylated and total c-JUN. **d**, **e** Viability of SUP-B15 and a CML blast crisis cell line K562 cells treated with various concentrations of JNK-IN-8 (**d**) or SP600125 (**e**) for 48 h was measured by the CellTiter Glo assay. Normalized cell proliferation was presented and compound’s IC50 value calculated. **f**, **g** Viability of CD19^+^ B lymphocytes isolated from normal and BCR/ABL^p190^ bone marrow transduction and transplantation mice treated with various concentrations of JNK-IN-8 (**f**) or SP600125 (**g**) for 48 h was measured by the CellTiter Glo assay. Normalized cell proliferation was presented and compound’s IC50 value calculated. **h** The nucleated bone marrow cells (NBMC) from a healthy donor and primary BM cells isolated from 6 patients with Ph^+^ B-ALL were treated with different concentrations of JNK-IN-8 for 48 h. Cell viability was measured by the CellTiter Glo assay. Data are presented as mean ± SD, and *P* values were calculated using Student’s *t* test. *P < 0.05, **P < 0.01, and ***P < 0.001

We then tested the effect of JNK inhibition on Ph^+^ B-ALL cells. We found that knockdown of JNK by short hairpin RNA (shRNA) targeting *JNK* in SUP-B15 cells (Fig. 2a) significantly inhibited Ph^+^ B-ALL cell proliferation (Fig. 2b). We further tested the effect of JNK inhibition on Ph^+^ B-ALL cells using two known small-molecule JNK inhibitors, JNK-IN-8 and SP600125. Inhibition of JNK kinase activity by either JNK-IN-8 or SP600125 markedly reduced the phosphorylation of JNK substrate c-JUN (Fig. 2c). These JNK inhibitors inhibited SUP-B15 cell proliferation in a dose-response manner (Fig. 2d, e). Interestingly, inhibition of JNK kinase activity by JNK-IN-8 and SP600125 had no apparent effect on CML blast phase cell line K562 (Fig. 2d, e).

We also examined the effect of JNK kinase inhibitors JNK-IN-8 and SP600125 on mouse B-ALL cells from the above BCR-ABL^P190^-transduced mouse model. Compared to CD19^+^ normal B cells, mouse BCR-ABL^+^ B-ALL cells were significantly more susceptible to the treatment of the two JNK inhibitors (Fig. 2f, g). Due to the better potency and target specificity [28], we use JNK-IN-8 as single or combined regimen in the following experiments.

We further examined the effect of JNK-IN-8 on six patient-derived Ph^+^ B-ALL cells. We found that the primary B-ALL cells were also significantly more susceptible to the treatment of JNK-IN-8 than normal bone marrow cells (NBMCs) (Fig. 2h). The detailed information of Ph^+^ B-ALL patients is shown in Supplementary table 2.

JNK has been shown to play an important role in initiation/development of B-ALL, as *Mapk8* deficiency delays the onset of leukemia [21]. Here we demonstrate that JNK also plays a role in the maintenance of human Ph^+^ B-ALL and that JNK inhibition could serve as a therapeutic intervention strategy for Ph^+^ B-ALL.

### JNK inhibition cooperates with dasatinib to reduce viability of Ph^+^ B-ALL cells

**Fig. 3:**
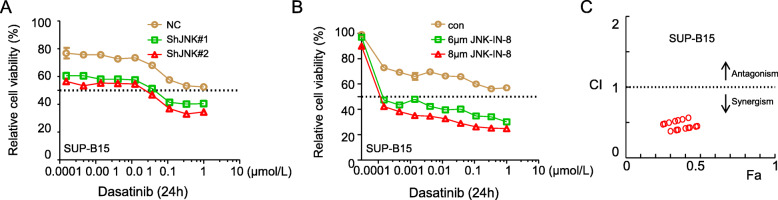
JNK inhibition cooperates with dasatinib in killing Ph^+^ B-ALL cells. **a** SUP-B15 cells expressing ShNC, ShJNK#1, or ShJNK#2 were treated with different concentrations of dasatinb for 24 h. Viability of these cells was then measured by the CellTiter Glo assay. **b** SUP-B15 cells were treated with indicated concentrations of dasatinib combined with or without JNK-IN-8 (6 μmol/L and 8 μmol/L) for 24 h. Viability of these cells was measured by the CellTiter Glo assay. **c** “Combination index” (CI) values were calculated at each concentration of JNK-IN-8 based on data in **b** and plotted, Fractional inhibition abbreviated as Fa. Data are presented as mean ± SD

To evaluate the potential cooperative effect of JNK inhibitor and BCR-ABL TKI, we first tested the effect of JNK knockdown by shRNA on viability of Ph^+^ B-ALL cells in the presence of various concentrations of dasatinib. Figure 3a shows that low concentrations of dasatinib has a moderate inhibitory effect on viability of SUP-B15 cells transfected with scrambled shRNA. JNK knockdown by either one of the two shRNA targeting JNK significantly enhanced dasatinib’s inhibitory effect on viability of SUP-B15 cells. Similarly, co-treatment with JNK-IN-8 significantly enhanced dasatinib’s inhibitory effect on viability of SUP-B15 cells (Fig. 3b).

To quantitatively analyze the synergistic effect of the two drugs, the combination index (CI) values at each combination dose were calculated by Calcusyn. Figure 3c shows a strong synergistic effect between JNK-IN-8 and dasatinib (Fig. 3c). The detailed information of CI value calculations is listed in Supplementary table 3.

**Fig. 4:**
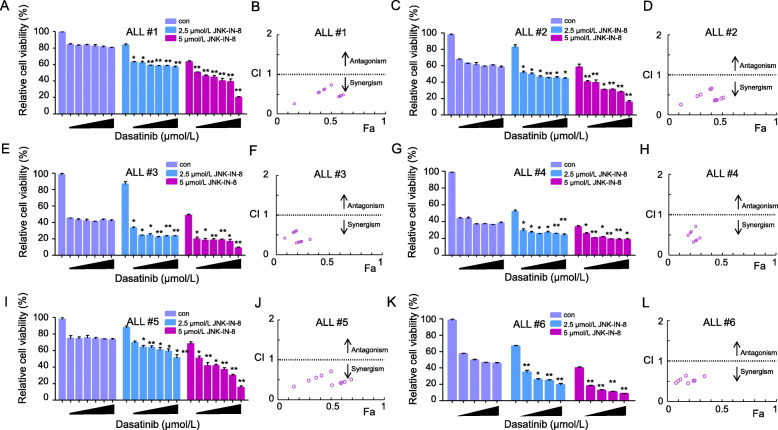
JNK-IN-8 cooperates with dasatinib in killing primary Ph^+^ B-ALL cells. **a**, **c**, **e**, **g**, **i**, and **k** Primary bone marrow cells isolated from 6 Ph^+^ B-ALL patients were treated with dasatinib (at the concentrations of 0, 0.125, 0.25, 0.5, 1, and 2 μmol/L), JNK-IN-8 (2.5, 5 μmol/L ) or both in combination for 48 h. Cell viability was measured by the CellTiter Glo assay. Data are presented as mean ± SD; *P* values were calculated by the comparison between the combination group and JNK-IN-8 or dasatinib alone group. *P < 0.05, **P < 0.01. **b**, **d**, **f**, **h**, **j**, and **l** CI values were calculated based on data from **a**, **c**, **e**, **g**, **i**, and **k**, respectively, and plotted. Fractional inhibition abbreviated as Fa

We further tested the combination treatment of JNK-IN-8 and dasatinib in patient-derived Ph^+^ B-ALL cells. Consistent with the result in SUP-B15 cells, combination treatment of JNK-IN-8 and dasatinib significantly reduced cell viability of Ph^+^ B-ALL primary cells when compared to the treatment with dasatinib alone (Fig. 4a, c, e, g, i, and k). The combination index calculation shows JNK-IN-8 has a strong synergistic effect with dasatinib at all doses tested (Fig. 4b, d, f, h, j, and l). The detailed information of CI value calculations is listed in Supplementary table 3.

### The combination treatment with JNK-IN-8 and dasatinib significantly prolongs life of BCR-ABL^+^ B-ALL mice

To confirm the therapeutic effect of JNK inhibitor combined with dasatinib on BCR-ABL^+^ B-ALL in vivo, we tested the efficacy of JNK inhibitor plus dasatinib in the BCR-ABL^P190^ bone marrow transduction and transplantation mouse model described above. Starting from the day 10 post bone marrow transplantation (BMT), recipient mice received daily treatment with vehicle, dasatinib (2 mg/kg), JNK-IN-8 (20 mg/kg), and the combination of dasatinib plus JNK-IN-8 for 18 consecutive days, respectively.

**Fig. 5:**
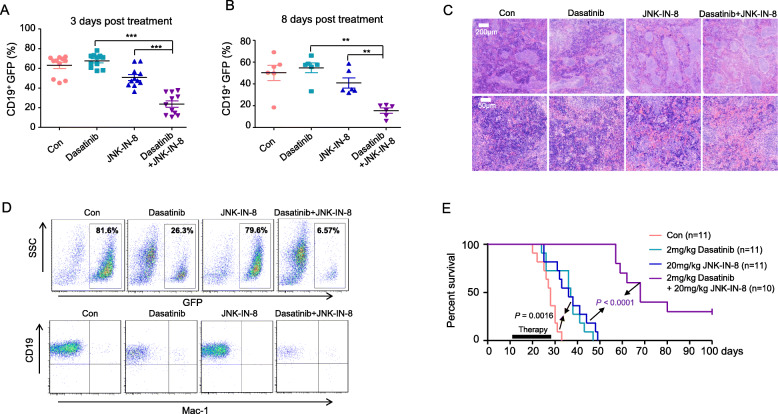
The combination treatment with JNK-IN-8 and dasatinib significantly prolongs the life of BCR/ABL^+^ B-ALL mice. **a** Percentage of GFP-positive CD19^+^ B lymphocytes in peripheral blood (PB) from BCR/ABL^p190^ bone marrow transduction and transplantation mice treated with vehicle, dasatinib (2 mg/kg), JNK-IN-8 (20 mg/kg) alone, or both in combination QD, respectively, for 3 days. **b** Percentage of GFP-positive CD19^+^ B lymphocytes in PB from BCR/ABL^p190^ bone marrow transduction and transplantation mice treated with compounds as described in **a** for 8 days. **c** Hematoxylin and eosin staining of spleens from BCR/ABL^p190^ bone marrow transduction and transplantation mice treated with compounds as described in **a** for 10 days. Scale bars in the upper and lower panel are 200 μm and 50 μm, respectively. **d** Flow cytometry analysis of bone marrow cells from mice as described in C. **e** The Kaplan-Meier survival curves of BCR/ABL^p190^ bone marrow transduction and transplantation mice continuously administrated with vehicle, dasatinib (2 mg/kg), JNK-IN-8 (20 mg/kg) alone, or both drugs in combination QD for 18 days. *P* values were calculated by log-rank test and shown

Interestingly, the combination treatment markedly decreased the percentage of CD19^+^ GFP cells circulating in the peripheral blood just after 3 days drug administration (Fig. 5a). Similarly, 8 days treatment later, the combination treatment further lowered the percentage of leukemia cells in the peripheral blood, whereas dasatinib or JNK-IN-8 alone treatment only had a moderate effect (Fig. 5b).

After 10 days of treatment, we randomly sacrificed one recipient mouse from each group and conducted an autopsy to evaluate the leukemia burden. The hematoxylin and eosin (H&E) staining data showed that the combination treatment significantly inhibited the infiltration of leukemia cells in the spleen, while diseased mouse treated with dasatinib or JNK-IN-8 alone had more severe leukemia cell infiltration in the spleen (Fig. 5c). Furthermore, FACS analysis of bone marrow cells showed that GFP positive B lymphoid leukemia cells were dramatically reduced upon dasatinib treatment (26.3%), as compared to the untreated group (81.6%). Although JNK-IN-8 alone treatment had little effect in reducing leukemia cells in the bone marrow, leukemia cells were nearly depleted in the bone marrow of mouse with the dasatinib plus JNK-IN-8 combination treatment (6.57%).

As expected, dasatinib treatment under the dosage used prolonged life of mice with BCR-ABL^+^ B-ALL (Fig. 5e). Interestingly, treatment with JNK-IN-8 alone also prolonged life of mice with BCR-ABL^+^ B-ALL (Fig. 5e). But the effect of combination treatment with dasatinib and JNK-IN-8 is much more than either inhibitor treatment alone (Fig. 5e). Notably, there were still three mice alive in the combination treatment group at the end of observation period (Fig. 5e). These results indicate that JNK inhibition could cooperate with BCR-ABL TKI to suppress BCR-ABL^+^ B-ALL.

### Synergistic effect of dasatinib and JNK-IN-8 in downregulating c-MYC expression

Previous studies have shown that JNK inhibitors exerted their therapeutic effect in tumor through regulating the downstream effectors, such as c-MYC [29, 30] and MCL-1 [31]. However, how JNK inhibitors work in Ph^+^ B-ALL cells is unclear. To explore how the JNK inhibitor affects Ph^+^ B-ALL cells, we treated the SUP-B15 cells with JNK-IN-8 and examined the expression of anti-apoptotic proteins MCL-1, BCL-XL, and BCL-2, as well as c-MYC. We found that the expression of MCL-1 was decreased in response to the treatment of JNK-IN-8, but only at higher concentrations (Supplementary Fig. S3a). The JNK-IN-8 treatment did not affect the expression of BCL-XL and BCL-2. On the contrary, the JNK-IN-8 treatment leads to a dramatic decrease of c-MYC expression (Supplementary Fig. S3a). Consistently, JNK knocking down by shRNAs also significantly reduced c-MYC expression (Supplementary Fig. S3b).

It has been shown that c-MYC plays a critical role in the proliferation of Ph^+^ B-ALL cells [32]. To confirm the c-MYC function in Ph^+^ B-ALL cells, we treated SUP-B15 cells with the BRD4 inhibitor JQ-1 [33], a potent suppressor for c-MYC expression. We found that JQ-1 significantly decreased c-MYC expression and inhibited SUP-B15 cells proliferation in a dose-dependent manner (Supplementary Fig. S3c, d and e). Like JNK inhibitor, JQ-1 had no apparent effect on the CML blast phase cell line K562 (Supplementary Fig. S3c and d). Consistent with the results in SUP-B15 cells, the JQ-1 treatment also inhibited the proliferation of primary B-ALL cells (Supplementary Fig. S3e). In contrast, JQ1 only inhibited the proliferation of normal bone marrow cell control at higher concentrations.

Interestingly, the dasatinib treatment also reduced c-MYC expression, but the effect was much more dramatic when used in combination with JNK-IN-8 (Supplementary Fig. S3f). Consistent with the synergy between JNK-IN-8 and dasatinib, JQ-1 also enhanced the effect of dasatinib in inhibiting the proliferation of SUP-B15 cells (Supplementary Fig. S3g). These results suggest that dasatinib and JNK-IN-8 suppress Ph^+^ B-ALL synergistically at least partially through downregulating the c-MYC expression.

## Discussion

The application of BCR-ABL tyrosine kinase inhibitor dasatinib in Ph^+^ B-ALL has largely improved the initial complete remission rate. However, many patients without secondary BCR-ABL mutations ended up with relapse after treatment [8, 34], implying the contribution of alternative survival pathways to the dasatinib resistance. Therefore, eradication of Ph^+^ B-ALL cells requires inhibition of additional targets beyond BCR-ABL.

In this study, we found BCR-ABL TKI dasatinib is unable to inhibit the abnormally activated JNK pathway in BCR-ABL^+^ B-ALL. Furthermore, the expression of p-JNK was increased in Ph^+^ B-ALL cells after treated with dasatinib. JNK inhibition by either genetic or chemical intervention could potently kill human BCR-ABL^+^ B-ALL cells. More importantly, JNK inhibition can effectively treat BCR-ABL^+^ B-ALL synergistically with dasatinib. These results indicate that JNK is a key signaling for dasatinib resistance in Ph^+^ B-ALL.

It is previously reported that JNK deficiency in mice delays the onset of B-ALL induced by BCR-ABL [21]. But the probability and feasibility of JNK-targeting therapy in BCR-ABL^+^ B-ALL was not unclear. Here we demonstrate that JNK plays an important role in the maintenance of Ph^+^ B-ALL and it is an effective target for combination therapy of BCR-ABL^+^ B-ALL together with BCR-ABL TKI dasatinib.

Side effects of dasatinib such as weight loss [35], cardiotoxicity [10], and pulmonary hypertension [11] are frequently encountered. In this study, we reduced the dose of dasatinib to 2 mg/kg QD instead of the standard dose (5 mg/kg QD). Our results show that the combined treatment with JNK-IN-8 and dasatinib exhibits a better therapeutic effect in prolonging the life of BCR-ABL^+^ B-ALL mice than that by either JNK inhibitor or dasatinib alone.

It was shown that JNK activation plays a crucial role in the induction of apoptosis of CML cells [36, 37]. Consistently, our data show that JNK inhibition has no therapeutic effect in CML cells. These findings indicate that JNK plays a context-dependent function in BCR-ABL^+^ B-ALL Vs. CML. The differential requirement of certain pathways in BCR-ABL^+^ B-ALL and CML has also been shown previously. As an example, Src kinases Lyn, Hck, and Fgr are required for BCR-ABL-induced B-ALL but not in CML [38]. In addition, the noncanonical Wnt pathway controlled by ɣ-catenin has recently been identified to be selectively required in the initiation and maintenance of Ph^+^ B-ALL cells but not in CML [32].

It has been shown that JNK activation also plays an important role in the survival of B cell lymphoma [30] and T cell acute lymphoblastic leukemia [39]. The significant therapeutic effects of JNK inhibitor in a B cell lymphoma mouse model was demonstrated [30]. A number of JNK inhibitors with various activity and selectivity to JNK kinases have been developed. JNK-IN-8 is a potent and specific JNK inhibitor that can covalently bind the Cys 116 residue of all three JNK kinases and inhibit them in an irreversible way [28]. In this study, we chose JNK-IN-8 to treat Ph^+^ B-ALL in combination with dasatinib in vivo. JNK-IN-8 showed therapeutic activity both alone and, to a much larger extent, in combination with dasatinib in treating BCR/ABL^+^ B-ALL.

It has been shown that JNK inhibitors exerted their therapeutic effect in tumor through regulating the downstream effectors, such as c-Myc [29, 30], and that c-MYC plays a critical role in the proliferation of Ph^+^ B-ALL cells [32]. Here we show that the JNK-IN-8 treatment or JNK knocking down dramatically downregulates the expression of c-MYC (Supplementary Fig. S3a). The BRD4 inhibitor JQ-1 that downregulates c-MYC expression and inhibits the proliferation of Ph^+^ B-ALL cells in a dose-dependent manner (Supplementary Fig. S3c, d and e). Interestingly, the dasatinib treatment also reduced c-MYC expression, but the effect was much more dramatic when used in combination with JNK-IN-8 (Supplementary Fig. S3f). Consistent with the synergy between JNK-IN-8 and dasatinib, JQ-1 also enhanced the effect of dasatinib in inhibiting the proliferation of SUP-B15 (Supplementary Fig. S3g). These results suggest that dasatinib and JNK-IN-8 suppress Ph^+^ B-ALL synergistically at least partially through downregulating the c-MYC expression.

In conclusion, we demonstrate that targeting JNK signaling pathway could synergistically treat Ph^+^ B-ALL with BCR-ABL TKI, providing a new therapeutic strategy for Ph^+^ B-ALL.

## Supplementary information

**Additional file 1: Table S1.** JNK kinase activity.

**Additional file 2 Supplementary Table S2.** Primary human Ph^+^ B-ALL samples. **Supplementary Table S3.** CI values information.

**Additional file 3: Figure S1.** The effect of dasatinib on CML cell line and primary Ph^+^ B-ALL cells. **Figure S2.** Preparation of mouse BCR-ABL^+^ B-ALL cells and KiNativ profiling of dasatinib and imatinib. **Figure S3.** Dasatinib and JNK inhibitor suppress c-MYC expression synergistically in Ph^+^ B-ALL cells.

## Data Availability

All data generated or analyzed during this study are included in this published article and its supplementary information files.

## Abbreviations

Ph^+^ B-ALL: Philadelphia chromosome-positive acute B-lymphoblastic leukemia
CML: Chronic myelogenous leukemia
TKI: ABL tyrosine kinase inhibitor
JNK: c-JUN N-terminal kinase
MAPK: Mitogen-activated protein kinase
ERK: Extracellular signal-regulated kinase
STAT: Signal transducer and activator of transcription
BMCs: Bone marrow cells
NBMCs: Normal bone marrow cells
shRNAs: Short hairpin RNAs
BMT: Bone marrow transplantation
FITC: Fluorescein isothiocyanate
GFP: Green fluorescent protein
CI: Combination index
Fa: Fractional inhibition
H&E: Hematoxylin and eosin
SDS: Sodium dodecyl sulfate
