# Supplementary material for: Combination therapy of BCR-ABL-positive B cell acute lymphoblastic leukemia by tyrosine kinase inhibitor dasatinib and c-JUN N-terminal kinase inhibition
Source: J Hematol Oncol. 2020 Jun 18;13:80. doi: 10.1186/s13045-020-00912-3 (PMC7302132; doi:10.1186/s13045-020-00912-3)
Supplement: Supplementary file 2 — Additional file 2 Supplementary Table S2. Primary human Ph+ B-ALL samples. Supplementary Table S3. CI values information. [file 13045_2020_912_MOESM2_ESM.docx]

**Supplementary Table S2. Primary human Ph^+^ B-ALL samples**

| ID sample | BCR/ABL1 | Age | Gender | Source | TKI therapy | Additional genetic abnormalities |
| --- | --- | --- | --- | --- | --- | --- |
| ALL #1 | P190 P210 | 29 | male | BM | No | No mutation found |
| ALL #2 | P190 | 42 | female | BM | No | No mutation found |
| ALL #3 | P190 | 44 | male | BM | No | IGH FR1-JH, IGH FR2-JH, TCRBVβ-Jβ gene rearrangement, DNMT3A-PHD gene mutation |
| ALL #4 | P210 | 81 | female | BM | No | No mutation found |
| ALL #5 | P190 | 72 | male | BM | No | IGH-DH-JH gene rearrangement |
| ALL #6 | P210 | 40 | male | BM | No | No mutation found |

BM: bone marrow

**Supplementary Table S3. CI values information**

| SUP-B15 | | | |
| --- | --- | --- | --- |
| dasa(µmol/L) | JNK-IN-8(µmol/L) | Fa | CI |
| 0.00015 | 6 | 0.47 | 0.45 |
| 0.00015 | 8 | 0.42 | 0.57 |
| 0.00046 | 6 | 0.43 | 0.43 |
| 0.00046 | 8 | 0.38 | 0.55 |
| 0.0014 | 6 | 0.48 | 0.45 |
| 0.0014 | 8 | 0.35 | 0.53 |
| 0.004 | 6 | 0.42 | 0.43 |
| 0.004 | 8 | 0.35 | 0.53 |
| 0.012 | 6 | 0.4 | 0.42 |
| 0.012 | 8 | 0.33 | 0.52 |
| 0.037 | 6 | 0.4 | 0.42 |
| 0.037 | 8 | 0.29 | 0.50 |
| 0.111 | 6 | 0.35 | 0.40 |
| 0.111 | 8 | 0.26 | 0.48 |
| 0.333 | 6 | 0.34 | 0.39 |
| 0.333 | 8 | 0.25 | 0.48 |
| 1 | 6 | 0.3 | 0.38 |
| 1 | 8 | 0.25 | 0.48 |

| ALL #1 | | | | ALL #2 | | | |
| --- | --- | --- | --- | --- | --- | --- | --- |
| dasa(µmol/L) | JNK-IN-8(µmol/L) | Fa | CI | dasa(µmol/L) | JNK-IN-8(µmol/L) | Fa | CI |
| 0.125 | 2.5 | 0.59 | 0.46 | 0.125 | 2.5 | 0.51 | 0.43 |
| 0.125 | 5 | 0.5 | 0.73 | 0.125 | 5 | 0.4 | 0.66 |
| 0.25 | 5 | 0.43 | 0.61 | 0.25 | 2.5 | 0.49 | 0.41 |
| 0.25 | 2.5 | 0.61 | 0.49 | 0.25 | 5 | 0.39 | 0.64 |
| 0.5 | 2.5 | 0.58 | 0.45 | 0.5 | 2.5 | 0.45 | 0.37 |
| 0.5 | 5 | 0.44 | 0.63 | 0.5 | 5 | 0.3 | 0.51 |
| 1 | 2.5 | 0.58 | 0.45 | 1 | 2.5 | 0.45 | 0.37 |
| 1 | 5 | 0.39 | 0.55 | 1 | 5 | 0.3 | 0.51 |
| 2 | 2.5 | 0.57 | 0.44 | 2 | 2.5 | 0.44 | 0.37 |
| 2 | 5 | 0.38 | 0.54 | 2 | 5 | 0.27 | 0.47 |
| 4 | 2.5 | 0.58 | 0.45 | 4 | 2.5 | 0.43 | 0.36 |
| 4 | 5 | 0.16 | 0.26 | 4 | 5 | 0.12 | 0.26 |
| ALL #3 | | | | ALL #4 | | | |
| dasa(µmol/L) | JNK-IN-8(µmol/L) | Fa | CI | dasa(µmol/L) | JNK-IN-8(µmol/L) | Fa | CI |
| 0.125 | 2.5 | 0.32 | 0.39 | 0.125 | 2.5 | 0.29 | 0.42 |
| 0.125 | 5 | 0.19 | 0.61 | 0.125 | 5 | 0.25 | 0.71 |
| 0.25 | 2.5 | 0.24 | 0.34 | 0.25 | 2.5 | 0.26 | 0.37 |
| 0.25 | 5 | 0.17 | 0.58 | 0.25 | 5 | 0.2 | 0.55 |
| 0.5 | 2.5 | 0.19 | 0.30 | 0.5 | 2.5 | 0.25 | 0.36 |
| 0.5 | 5 | 0.17 | 0.58 | 0.5 | 5 | 0.21 | 0.58 |
| 1 | 2.5 | 0.21 | 0.32 | 1 | 2.5 | 0.26 | 0.37 |
| 1 | 5 | 0.18 | 0.59 | 1 | 5 | 0.18 | 0.49 |
| 2 | 2.5 | 0.22 | 0.32 | 2 | 2.5 | 0.25 | 0.36 |
| 2 | 5 | 0.16 | 0.56 | 2 | 5 | 0.18 | 0.49 |
| 4 | 2.5 | 0.23 | 0.33 | 4 | 2.5 | 0.23 | 0.32 |
| 4 | 5 | 0.08 | 0.42 | 4 | 5 | 0.18 | 0.49 |
| ALL #5 | | | | ALL #6 | | | |
| dasa(µmol/L) | JNK-IN-8(µmol/L) | Fa | CI | dasa(µmol/L) | JNK-IN-8(µmol/L) | Fa | CI |
| 0.125 | 2.5 | 0.69 | 0.51 | 0.125 | 2.5 | 0.34 | 0.63 |
| 0.125 | 5 | 0.5 | 0.72 | 0.125 | 5 | 0.17 | 0.64 |
| 0.25 | 2.5 | 0.59 | 0.42 | 0.25 | 2.5 | 0.25 | 0.52 |
| 0.25 | 5 | 0.41 | 0.61 | 0.25 | 5 | 0.12 | 0.55 |
| 0.5 | 2.5 | 0.63 | 0.45 | 0.5 | 2.5 | 0.24 | 0.51 |
| 0.5 | 5 | 0.41 | 0.61 | 0.5 | 5 | 0.1 | 0.51 |
| 1 | 2.5 | 0.6 | 0.43 | 1 | 2.5 | 0.19 | 0.45 |
| 1 | 5 | 0.36 | 0.55 | 1 | 5 | 0.08 | 0.46 |
| 2 | 2.5 | 0.59 | 0.42 |  |  |  |  |
| 2 | 5 | 0.29 | 0.48 |  |  |  |  |
| 4 | 2.5 | 0.51 | 0.36 |  |  |  |  |
| 4 | 5 | 0.15 | 0.33 |  |  |  |  |

dasa = dasatinib; Fa = Fractional inhibition; CI = Combination index
