# Supplementary material for: Combination therapy of BCR-ABL-positive B cell acute lymphoblastic leukemia by tyrosine kinase inhibitor dasatinib and c-JUN N-terminal kinase inhibition
Source: J Hematol Oncol. 2020 Jun 18;13:80. doi: 10.1186/s13045-020-00912-3 (PMC7302132; doi:10.1186/s13045-020-00912-3)
Supplement: Supplementary file 3 — Additional file 3: Figure S1. The effect of dasatinib on CML cell line and primary Ph+ B-ALL cells. Figure S2. Preparation of mouse BCR-ABL+ B-ALL cells and KiNativ profiling of dasatinib and imatinib. Figure S3. Dasatinib and JNK inhibitor suppress c-MYC expression synergistically in Ph+ B-ALL cells. [file 13045_2020_912_MOESM3_ESM.docx]

**Combination therapy of BCR-ABL-positive B Cell Acute Lymphoblastic Leukemia by Tyrosine Kinase Inhibitor Dasatinib and c-Jun N-terminal Kinase inhibition**

Xinhua Xiao^1^, Ping Liu^1^, Donghe Li^1^, Zhizhou Xia^1^, Peihong Wang^1^, Xiuli Zhang^1^, Mingzhu Liu^1^, Lujian Liao^2^, Bo Jiao^1^, Ruibao Ren^1,3^

1 State Key Laboratory for Medical Genomics, Shanghai Institute of Hematology, Collaborative Innovation Center of Hematology, National Research Center for translational Medicine, Ruijin Hospital, Shanghai Jiao Tong University School of Medicine, Shanghai, China.

2 Shanghai Key Laboratory of Regulatory Biology, and Shanghai Key Laboratory of Brain Functional Genomics, School of Life Sciences, East China Normal University, Shanghai, China.

3 Department of Biology, Brandeis University, Waltham, MA, USA.

Corresponding Authors: Ruibao Ren, E-mail: ren@brandeis.edu; Bo Jiao, E-mail: bjiao@sibs.ac.cn.

**Supplementary Figures**


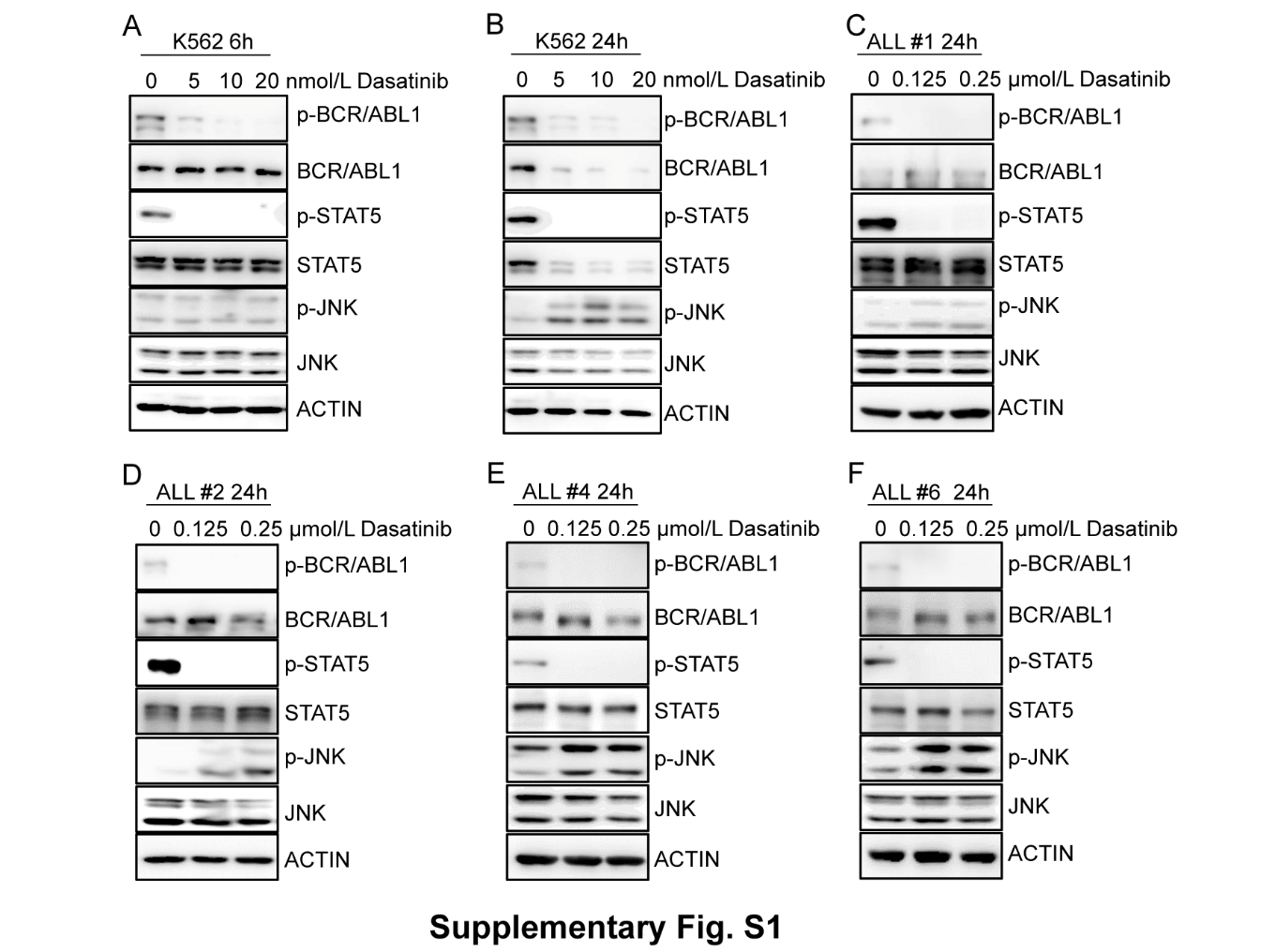


**Figure S1. The effect of dasatinib on CML cell line and primary Ph^+^ B-ALL cells.** K562 cells were treated with various concentrations of dasatinib for 6h (a) and 24h (b). Phosphorylated and total BCR/ABL, STAT5, and JNK were examined by western blot analysis using indicated antibodies. Actin was used as a loading control. (c, d, e, f) Primary BM cells isolated from four patients with Ph^+^ B-ALL were treated with different concentrations of dasatinib for 24h. Phosphorylated and total BCR/ABL, STAT5, and JNK were examined by western blot analysis using indicated antibodies. Actin was used as a loading control.


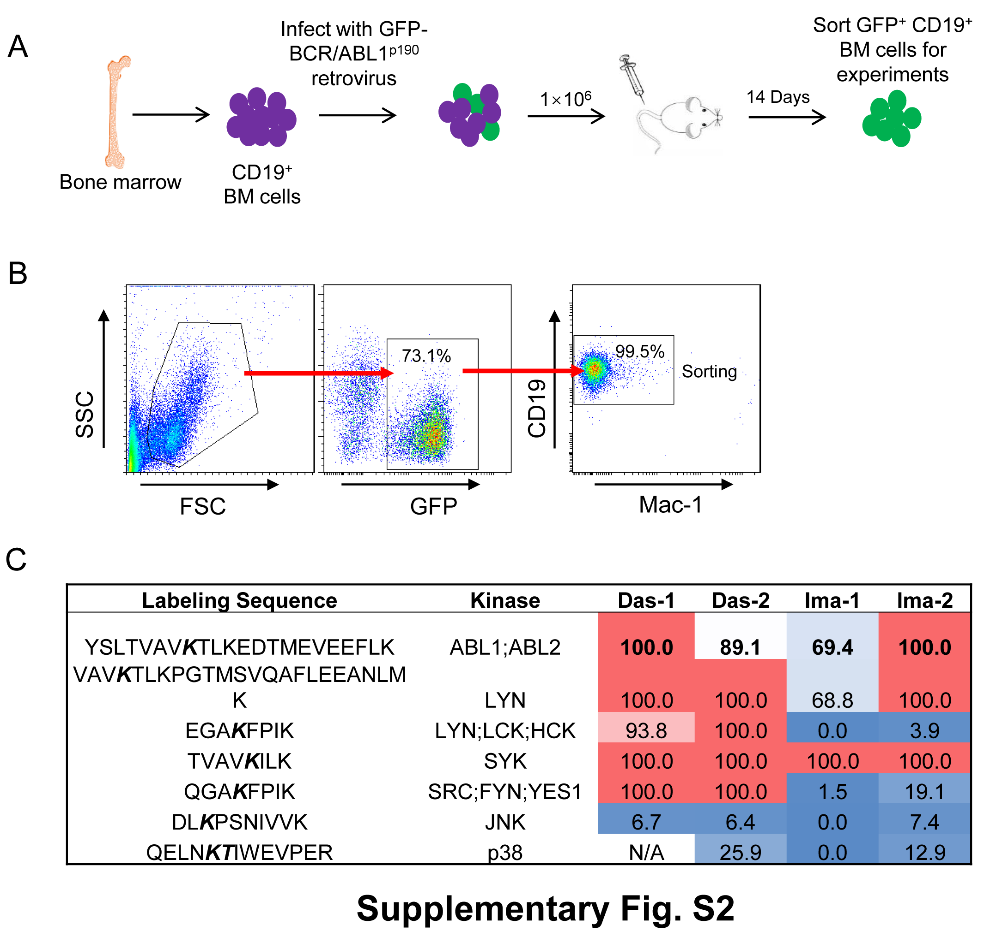


**Figure S2. Preparation of mouse BCR-ABL^+^ B-ALL cells and KiNativ profiling of dasatinib and imatinib.** (a) Schematic illustration of the generation of BCR/ABL^p190^ bone marrow transduction and transplantation mouse model. CD19^+^ B lymphocytes isolated from bone marrow of 6- to 8-week-old BALB/cByJ donor mice using magnetic-activated cell sorting technology were transduced with BCR/ABL^p190^ retroviruses, followed by transplantation of 10^6^ cells into each recipient mice. BCR/ABL^+^ B-ALL cells were isolated by FACS 14 days later. (b) GFP-positive CD19^+^ B lymphocytes were sorted from BM of BCR/ABL^p190^ bone marrow transduction and transplantation mice using BD FACSAria^TM^ Cell Sorter. Gating for cell sorting are shown. (c) Selected KiNativ profiling data from SUP-B15 cells treated with 0.3 μmol/L dasatinib or imatinib for 2 hours. Percent inhibition to indicated kinases are shown. Strong binding of inhibitors are highlighted by red color, weak binders are shaded with blue color.


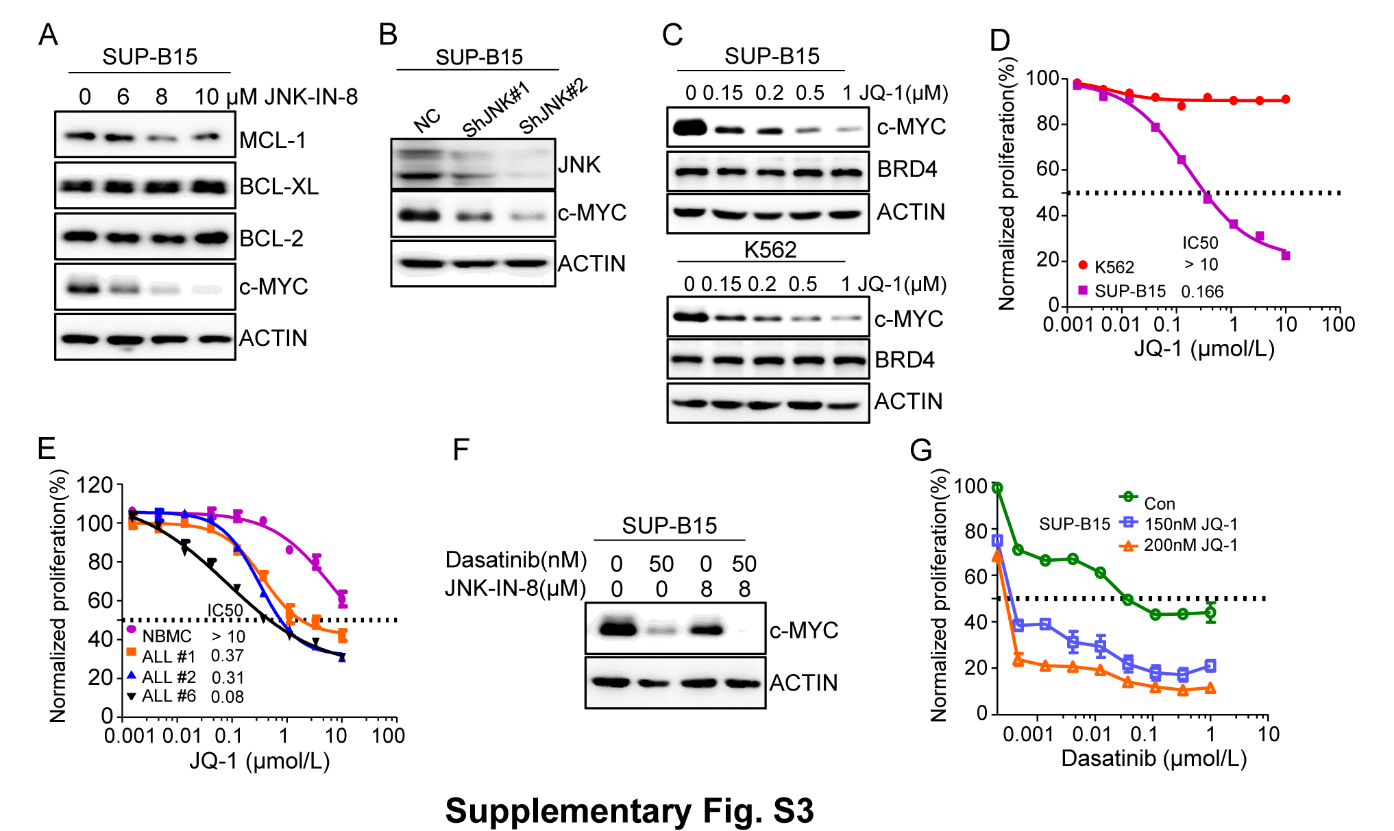


**Figure S3. Dasatinib and JNK inhibitor suppress c-MYC expression synergistically in Ph^+^ B-ALL cells.** (a) SUP-B15 cells were treated with various concentrations of JNK-IN-8 for 12h. The expression of MCL-1, BCL-XL, BCL-2 and c-MYC were examined by western blot analysis. Actin was used as a loading control. (b) The expression of JNK and c-MYC in SUP-B15 cells expressing ShNC, ShJNK#1 or ShJNK#2 were detected by western blot analysis. Actin was used as a loading control. (c) SUP-B15 and K562 cells were treated with BRD4 inhibitor JQ-1 for 6h. The expression of c-MYC and BRD4 was examined by western blot analysis. (d) Normalized cell proliferation of SUP-B15 and K562 cells treated with various concentrations of JQ-1 for 48h was measured by the CellTiter Glo assay. (e) The nucleated bone marrow cells (NBMC) from a healthy donor and three primary BM cells isolated from patients with Ph^+^ B-ALL were treated with different concentrations of JQ-1 for 48h. The normalized cell proliferation of these cells was then measured by the CellTiter Glo assay. (f) SUP-B15 cells were treated with the indicated concentrations of dasatinib or JNK-IN-8 for 6h. The expression of c-MYC was analyzed by western blot analysis. Actin was used as a loading control. (g) SUP-B15 cells were treated with various concentrations of dasatinib with or without indicated concentrations of JQ-1 for 48h. Normalized cell proliferation of these cells was measured by the CellTiter Glo assay.
